# Granuloma formation after repeated episodes of peritoneal dialysis catheter–related infection, a case report

**DOI:** 10.1186/s12882-023-03230-1

**Published:** 2023-06-16

**Authors:** Fang Cao, Li Zhang, Yiping Ruan, Miao Lin, Fuyuan Hong

**Affiliations:** Department of Nephrology, Provincial Clinical College, Fujian Medical University, Fujian Provincial Hospital, 350001 Fuzhou, China

**Keywords:** Granuloma formation, Dialysis, Catheter–related infection, Debridement

## Abstract

**Background:**

Peritoneal catheter related infection is one of the main complications and the major cause of technical failure of peritoneal dialysis (PD) treatment. However, PD catheter tunnel infection can be difficult to diagnosis and resolve. We presented a rare case in which a granuloma formation after repeated episodes of peritoneal dialysis catheter–related infection.

**Case presentation:**

A 53-year-old female patient with kidney failure due to chronic glomerulonephritis treated with peritoneal dialysis for 7 years. The patient had repeated exit-site and tunnel inflammation, and repeated suboptimal courses of antibiotics. She switched to hemodialysis after 6 years in a local hospital without the peritoneal dialysis catheter being removed. The patient complained of an abdominal wall mass that lasted for several months. She was admitted to the Department of surgery to undergo mass resection. The resected tissue of the abdominal wall mass was sent for pathological examination. The result showed foreign body granuloma with necrosis and abscess formation. After the surgery, the infection did not recur.

**Conclusions:**

The following key points can be learned from this case: 1. It is important to strengthen patient follow-up. 2.The PD catheter should be removed as early as possible in patients who do not need long-term PD, especially in patients with a history of exit-site and tunnel infections. 3. For patients presenting abnormal subcutaneous mass, attention should be paid to the possibility of the granuloma formation of infected Dacron cuffs of the PD catheter. If catheter infection occurs repeatedly, catheter removal and debridement should be considered.

## Introduction

Peritoneal dialysis (PD) is being increasingly used as the preferred renal replacement therapy by more patients with end stage renal disease (ESRD). We presented a case of granuloma formation after repeated episodes of peritoneal dialysis catheter–related infection. The abdominal wall mass turned out to be the Dacron cuffs wrapped in tissue due to granuloma formation, surrounded by protruding giant cells, inflammatory cell infiltration and fibrotic tissue. The Dacron cuffs of the PD catheter are made of polyester fiber, which will cause moderate inflammatory responses and fibroblast proliferation. The polyester cuffs on peritoneal catheters are designed to instigate an inflammatory response leading to fibrosis, that helps anchor the catheter within the exit site, reducing the risk of catheter dislodgment. However, this same process can be counterproductive in patients with repeated infections, leading to chronic inflammation and foreign body granuloma formation. In patients who developed repeated episodes of tunnel and catheter-related infection, the possibility of infectious granuloma formation should be considered. The need for surgical intervention should be assessed.

## Background

PD is an effective renal replacement treatment for end-stage renal disease (ESRD) allowing patients to perform self-assisted dialysis at home [1]. However, if the complications are not handled in a timely manner, treatment failure may occur [2]. Infection is one of the primary complications of peritoneal dialysis. Catheter-related infections include exit-site and tunnel infections [3]. The purpose of the case report is to suggest that we should actively treat catheter infection to avoid repeated infection leading to granuloma formation and ultimately failure of peritoneal dialysis [4]. Another teaching point in the case report is timely removal of a PD catheter when no longer in use.

## Case presentation

A 53-year-old female patient with kidney failure due to chronic glomerulonephritis began peritoneal dialysis 7 years ago. A straight Tenckhoff catheter was inserted. She presented exit-site and tunnel infection one month after PD initiation. She was admitted to a local hospital. The discharge from the exit-site grew Staphylococcus aureus, which was susceptible to cephalosporins including cefazolin. Dressing change at the exit-site was performed and oral cefixime was used. The symptoms improved after 2 weeks. There was no peritonitis.

**Fig. 1:**
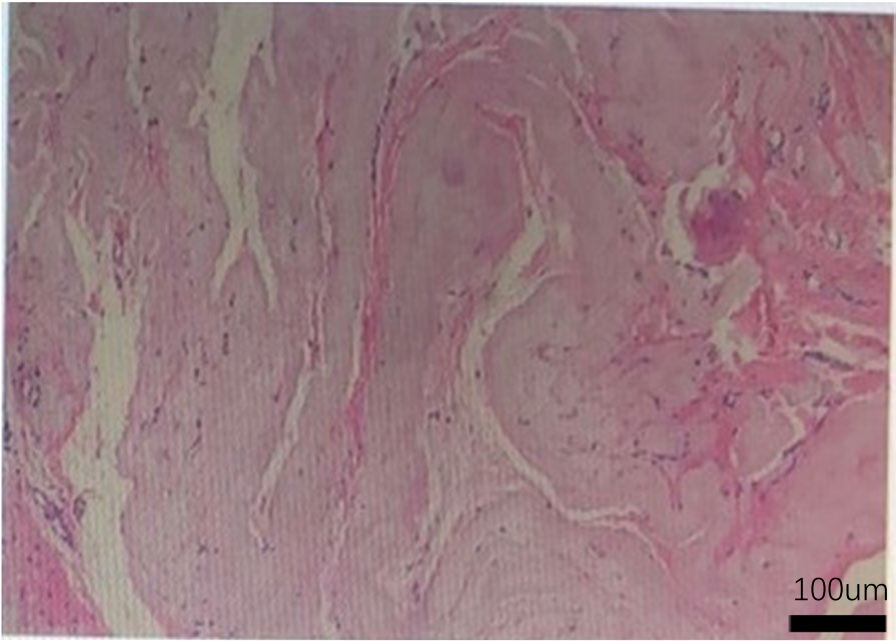
A haematoxylin- and eosin-stained section of abdominal wall mass (100 ×) in the examined tissues, a homogenous and foreign body without cellular components was observed under microscope; fibrous tissue hyperplasia of the surrounding cystic wall was present

**Fig. 2:**
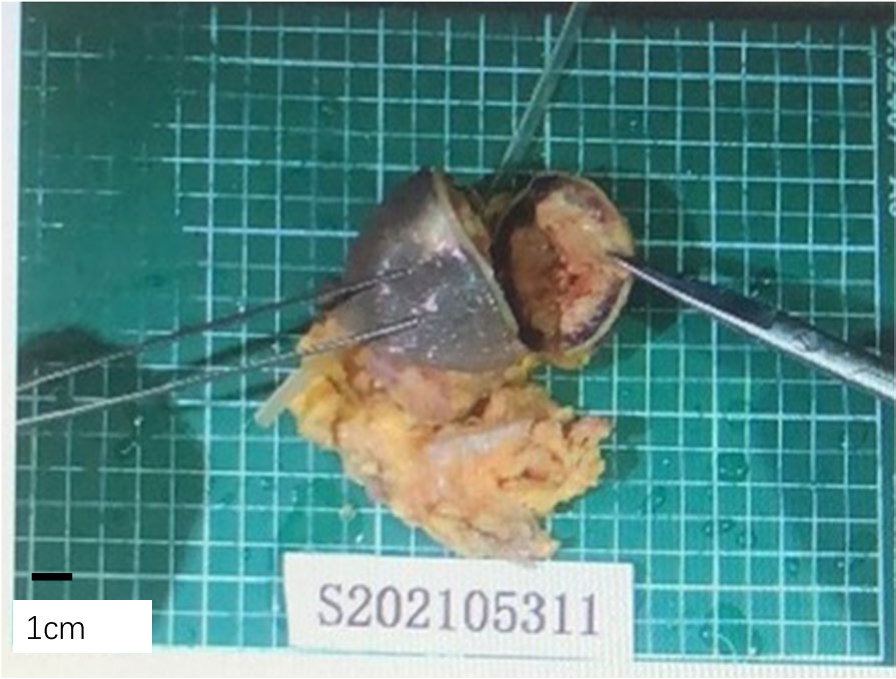
The resected tissue of the abdominal wall mass. There was a subcutaneous quasi-circular nodule at 0.5 cm from the base with a size of 6.5*6.0*5.0 cm and a thickness of 0.3–0.5 cm (including the capsule), and the capsule was complete. The nodule and the cuff of the peritoneal catheter were difficult to separate, being surrounded by a small amount of adipose tissue

In the following 7 years, the patient presented repeated episodes of exit-site and tunnel inflammation, and repeated suboptimal courses of antibiotics. She requested to convert to hemodialysis 1 year ago. However, despite elective discontinuation of peritoneal dialysis, with transition to hemodialysis, her peritoneal dialysis catheter was not removed. The patient complained of an abdominal wall mass that lasted for several months. On outpatient clinical evaluation, the mass was soft, fixed, slightly painful to touch, and was located along the tunnel and 2 cm from the catheter exit-site. She was admitted to the Department of Surgery to undergo mass resection. After local anesthetic, the anterior rectus sheath was sharp dissection; the intraperitoneal portion of the catheter is gently withdrawn from the peritoneal cavity. The abdominal mass was completely removed including the superficial cuff and the portion of the catheter. The resected tissue of the abdominal wall mass was sent for pathological examination. The pathological results were as follows: abdominal wall mass with a quasi-circular dermal nodule with a size of 10.0*9.0*8.0 cm; a skin area of 10.0*6.0 cm; and a broken surface area of 2.0*1.5 cm. In addition to the soft tissue, there was a subcutaneous quasi-circular nodule at 0.5 cm from the base with a size of 6.5*6.0*5.0 cm and a thickness of 0.3–0.5 cm (including the capsule), and the capsule was complete. The nodule and the cuff of the peritoneal catheter were difficult to separate, being surrounded by a small amount of adipose tissue (Fig. 1). The pathological diagnosis was as follows: in the examined tissues, a homogenous and foreign body without cellular components was observed under microscope; fibrous tissue hyperplasia of the surrounding cystic wall was present; foreign body granuloma with necrosis and abscess formation was observed (Fig. 2). After debridement, the infection did not recur, without any complications.

## Discussion and conclusion

The PD catheter used in this patient was a straight Tenckhoff catheter, which is the most widely used type of catheter in China and worldwide because it can reduce the incidence of catheter malfunction compared with curved catheters [4, 5]. The deep cuff of Tenckhoff catheters have been shown to be surrounded by protruding giant cells, foreign body granuloma, fibrosis and causing enveloped peritoneal sclerosis [6, 7]. A recent report showed tunnel infection induced by non-tuberculous *Mycobacterium* was associated with the formation of chroidal material composed of granulomas rather than pus [8]. In this case, the pathogen obtained from a culture was negative probably due to the suboptimally dosed use of antibiotics.

Here, we present a case in which a granuloma formation developed in a patient receiving PD after repeated episodes of peritoneal dialysis catheter–related infection. The following key points are learned from this case: 1. It is important to strengthen patient follow-up. For patients who did not visit the hospital for follow-up for more than 3 months, a specialty nurse should provide timely follow-up by the other manner, such as by telephone. 2.The PD catheter should be removed as early as possible in patients who do not need long-term PD, especially in patients with a history of exit-site and tunnel infections. 3. For patients presenting abnormal subcutaneous mass, attention should be paid to the possibility of the granuloma formation of infected Dacron cuffs of the PD catheter [3]. These patients may benefit from catheter removal and debridement.

## Data Availability

The datasets used during this case report are available from the corresponding author on reasonable request.

## References

[1] Parrish AR (2016). Advances in Chronic Kidney Disease. Int J Mol Sci.

[2] Boudville N, Cho Y, Equinox KL, Figueiredo AE, Hawley CM, Howard K, Johnson DW, Jose M, Lee A, Maley M, Moodie J, Pascoe EM, Steiner GZ, Tomlins M, Voss D, Chow J (2018). Teaching Peritoneal Dialysis in Australia: An Opportunity for Improvement. Nephrology Nephrology (Carlton).

[3] Octavian Mihalache, Horia Doran, Petronel Mustăţea, Florin Bobircă, Dragoş Georgescu, Andra Bîrligea, Alexandra Agache, Traian Pătraşcu.Surgical Complications of Peritoneal Dialysis. Chirurgia (Bucur).2018,113(5):611–624. 10.21614/chirurgia.113.5.611 30383988

[4] Chow KM, Wong SSM, Ng JKC, Cheng YL, Leung CB, Pang WF, Fung WWS, Szeto CC, Li PKT (2020). Straight Versus Coiled Peritoneal Dialysis Catheters: A Randomized Controlled Trial. Am J Kidney Dis.

[5] Ouyang CJ, Huang FX, Yang QQ, Jiang ZP, Chen W, Qiu Y, Yu XQ (2015). Comparing the Incidence of Catheter-Related Complications with Straight and Coiled Tenckhoff Catheters in Peritoneal Dialysis Patients-A Single-Center Prospective Randomized Trial. Perit Dial Int.

[6] Dimitriadis A, Antoniou S, Toliou T, Papadopoulos C (1990). Tissue reaction to deep cuff of Tenchoff’s catheter and peritonitis. Adv Perit Dial.

[7] Árnadóttir M, Gunnlaugur Jónasson J, Skúli Indridason Ó (2011). Encapsulating peritoneal sclerosis following a peritoneal foreign body reaction to Dacron fibres—a case report. NDT Plus.

[8] Noda Ryunosuke,Kamano Daisuke,Abe Tatsuki,Shinzawa Satoki,Takamatsu Manabu,Bae Yuan,Kobayashi Ryu,Yanagi Mai,Ishibashi Yoshitaka. Granuloma formation after peritoneal dialysis catheter-related infection by Mycobacterium chelonae. Kidney international,2021; 2021.03.040. 10.1016/j.kint.2021.03.040 34802569

